# Sensitive and Label-Free Colorimetric Detection of Glyphosate Based on the Suppression Peroxidase-Mimicking Activity of Cu(II) Ions

**DOI:** 10.3390/molecules28124630

**Published:** 2023-06-08

**Authors:** Qing Li, Yumeng Guo, Xiangyi He, Guangli Li

**Affiliations:** 1 College of Life Science and Chemistry, Hunan University of Technology, Zhuzhou 412007, China; 15874957110@163.com (Y.G.); 18818308194@163.com (X.H.); 2 State Key Laboratory of Chemo/Bio-Sensing and Chemometrics, College of Chemistry and Chemical Engineering, Hunan University, Changsha 410082, China

**Keywords:** label-free, colorimetric analysis, copper ions, peroxidase-mimicking activity, glyphosate

## Abstract

The sensitive and accurate determination of glyphosate (Glyp) is urgently demanded because it is closely correlated with human health and environmental safety. In this work, we proposed a sensitive and convenient colorimetric assay by employing copper ion peroxidases for the detection of Glyp in the environment. Free Cu(II) ions displayed high peroxidase activity and can catalytically oxidize the colorless 3,3′,5,5′-tetramethylbenzidine (TMB) into blue oxTMB, resulting in an obviously visible discoloration reaction. Once the Glyp is added, the ability of copper ions to mimic peroxidase can be largely suppressed because of the generation of Glyp–Cu^2+^ chelate. The favorable selectivity and sensitivity were demonstrated in the colorimetric analysis of Glyp. Furthermore, this rapid and sensitive method was successfully applied in the accurate and reliable determination of glyphosate in the real sample, holding promising applications in pesticide determination in the environment.

## 1. Introduction

Glyphosate was developed by Monsanto in the 1870s and has now become the world’s best-selling pesticide variety, used in over 100 countries and regions. Due to its advantages of broad spectrum, high efficiency, and low toxicity, Glyp is widely used for weeding in areas such as farmland, forests, highways, and lakes. In addition, Glyp is also used to improve feed quality, crop drying, and ripening [1]. Glyphosate can affect the biosynthesis of amino acids. Its main target is to inhibit the activity of 5-enolpyruvylshikimate-3-phosphate synthase (EPSP synthase), inhibit the biosynthesis of aromatic amino acids, hinder protein synthesis, and non-selective lead to plant death. Surfactants are essential adjuvants for glyphosate, which can induce glyphosate to be quickly absorbed by plants through stomata, avoiding rainwater leaching and significantly improving weed control efficiency [2]. Because of its unique properties, such as low cost and low toxicity to mammals compared to other herbicides, Glyp is widely used and uncontrolled. With the widespread use of glyphosate, residual Glyp in the soil is the main source of accumulation in crops. Glyp can also infiltrate with rainwater and irrigation water, contaminate groundwater, and remain and migrate in soil and water bodies, ultimately entering organisms through bioaccumulation or entering the human body through food chain interactions. Different research results indicate that the average half-life of glyphosate in freshwater systems is 10 weeks. After using glyphosate, laboratory, and field studies have found the presence of Glyp at a depth of 1 m, with a mass concentration of 2.2 mg/L [3]. Recent studies have shown that Glyp can inhibit the activity of acetylcholinesterase (AChE) and cause respiratory, myocardial, and neuromuscular dysfunction [4,5,6]. In 2015, the International Agency for Research on Cancer (IARC) released a report that listed glyphosate as a Class 2A carcinogen, which is likely to cause cancer in humans [4]. In its report, IARC stated that “limited evidence suggests that the herbicide glyphosate may cause non-Hodgkin’s lymphoma”. At the same time, there is sufficient evidence to prove that Glyp causes cancer in experimental animals. The United States Environmental Protection Agency set the maximum concentration of glyphosate in water as 700 μg/L, and the Canadian Health Guidelines for Drinking Water set the maximum acceptable concentration of Glyp in water as 280 μg/L [7]. Therefore, it is of great practical significance to develop a simple and accurate Glyp detection method with high sensitivity, strong specificity, and low cost.

Due to the increasingly serious threat of pesticide residues, a number of quantitative detection methods have been reported for the estimation of Glyp, including high-performance liquid chromatography [8], enzyme-linked immunosorbent assay (ELISA) [9,10], electrochemical method [11,12], and capillary electrophoresis (CE) [13]. Although these methods can achieve the accurate detection of Glyp, they also have some limitations, such as the time-consuming and laborious process of sample pretreatment and high experimental cost, which cannot meet the needs of facile and rapid detection [14]. Compared with these analytical methods, colorimetric detection technology has attracted wide attention because of its advantages of simple operation and low cost. Recently, researchers have developed some colorimetric detection methods for organophosphorus pesticides by utilizing their inhibitory effect on enzyme activity. For example, Jiang et al. developed a dual-mode detection method for organophosphorus pesticides based on the inhibition effect of Glyp on acetylcholinesterase activity by using labeled Au nanoparticles (AuNPs) [15]. Yan et al. [16] constructed a sensitive optical detection system for organophosphorus pesticides by using the inhibition of organophosphorus pesticides on the hydrolysis of acetylthiocholine catalyzed by Butyrylcholinesterase. However, the biological activity of natural enzymes is easily affected by environmental changes, which in turn affects the stability and reproducibility of detection methods. In recent years, nanomaterials with biomimetic enzyme activity (nanozymes) have received widespread attention [17]. Compared with natural enzymes, nanozymes have advantages such as stable physicochemical properties, easy preparation, modification, and purification [18]. Nanozymes include peroxidase-like enzymes (POD), catalase-like enzymes (CAT), and superoxide dismutase-like enzymes (SOD). Its components mainly include metal oxides, metal compounds, metal complexes, and carbon-based materials [19]. Metal compounds or metal complexes typically exhibit POD-like and CAT-like activities in nanozymes, while the phosphonate, carboxyl, and amino groups of Glyps can strongly coordinate with metal atoms, affecting the catalytic activity of nanozymes. Luo et al. [20] prepared a Co_3_O_4_ nanozyme with suitable POD-like activity, which was inhibited by binding with Glyp. Based on the inhibition of POD activity, the visual detection of Glyp was achieved with a detection limit of 0.7 mg/L. Tai et al. [21] prepared a copper nanocluster, and its POD-like activity was also inhibited by Glyp. The linear range for detecting Glyp was 0.02–2 mg/L, and the detection limit was 0.85 µg/L. Although these novel colorimetric sensors have made some progress in the detection of Glyp, there are still some drawbacks, such as long response time and complex synthesis processes of nanomaterials or nanozymes. Hence, it is imperative to develop simple, sensitive, and economical colorimetric strategies for Glyp determination.

As one of the most important transition metals, copper is an essential trace element in plants and animals and plays an important role in the life process. The active sites of many metalloenzymes and metalloproteins contain Cu(II) ions [22]. Copper compounds are often used as catalysts for the decomposition of peroxides due to their varied coordination structures and catalytic properties of activating small molecules. Copper sulfide nanorods, copper sulfide nanoparticles, and copper bivalent ions were reported to be used to simulate peroxidase and quantitatively detect hydrogen peroxide, glucose, and pyrophosphatase activity [23,24,25,26,27]. Compared with horseradish peroxidase (HRP) and nanozyme, copper ion peroxidases not only possess the characteristics of high sensitivity but also have the advantages of easy acquisition, no need for complex synthesis, easy storage, direct use without modification, simple operation, and low cost. These outstanding features inspired us to develop a colorimetric assay based on copper ion peroxidases for quantitative analysis of Glyp pesticides in the environment.

**Scheme 1:**
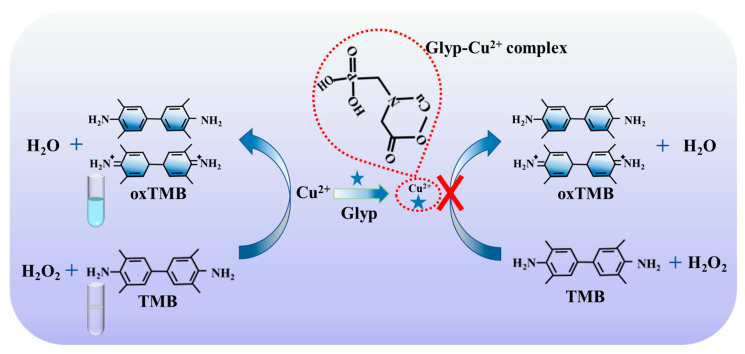
The principle of the colorimetric Glyp assay by using Cu(II) ions as a mimic peroxidase.

Herein, a colorimetric sensing method is developed for the detection of Glyp in the environment by utilizing Cu(II) ions. Scheme 1 illustrated the basic principles of Glyp colorimetric assay. When Glyp is absent, copper ion peroxidases catalyze the oxidation of TMB, producing a blue-colored oxTMB in an aqueous solution. When Glyp was added, the formation of Glyp–Cu^2+^ complexes inhibited the peroxide activity of Cu^2+^, resulting in the generation of less oxTMB and a hypochromic effect of the solution. Therefore, the concentration of Glyp can be detected according to the changes in the absorbance and color of the solution. Using Glyp to inhibit the peroxidase activity of Cu^2+^, a convenient and sensitive colorimetric method was established for the rapid quantitative determination of Glyp without using complicated instruments and protocol, thus providing a facile and reliable tool for the analysis of Glyp residues in the environment.

## 2. Results and Discussion

### 2.1. Cu(II) Ions Display Peroxidase Catalytic Activity

**Figure 1:**
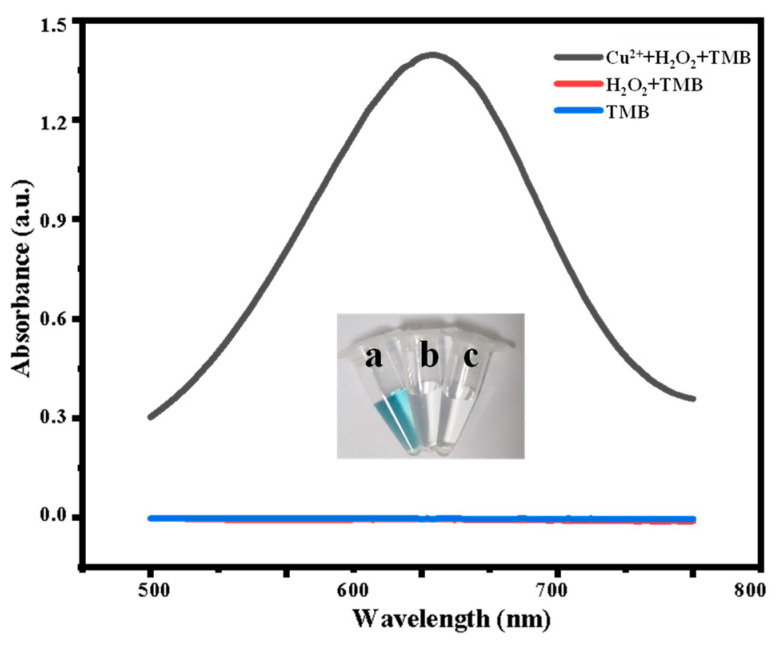
UV-vis spectra of Cu^2+^ + TMB + H_2_O_2_ (black line, a), TMB + H_2_O_2_ (red line, b), TMB (blue line, c). The inset presents the corresponding photograph.

In this study, we used Cu(II) ions as a potential peroxidase mimic. Compared with biological enzymes, the homogeneous nature of Cu(II) ions may make them have superior catalytic performance. TMB was employed as the chromogenic substrate to investigate the peroxidase-mimicking activity of Cu(II) ions. As shown in Figure 1, either TMB alone or the TMB–H_2_O_2_ system was colorless and merely displayed obvious absorbance at 653 nm. With the addition of Cu(II) ions into the TMB–H_2_O_2_ system, there was a significant absorbance enhancement at 653 nm, and the color of the mixture solution turned from colorless to blue. Such a comparison implies that the Cu(II) ions exhibit peroxidase activity properties.

**Figure 2:**
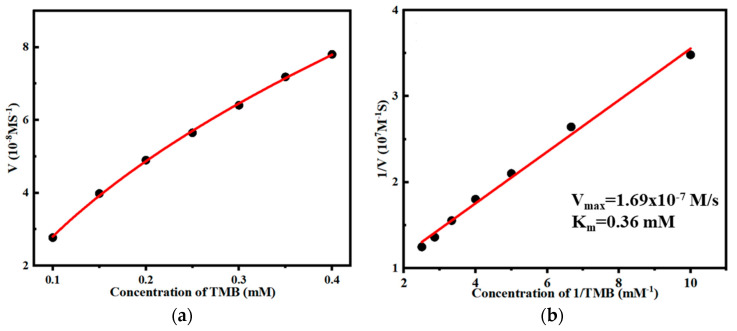

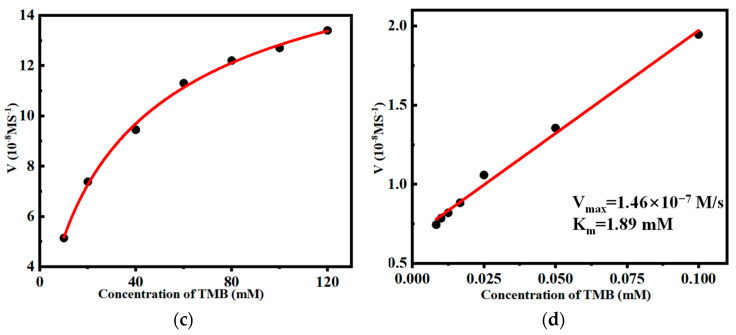
Steady-state kinetic analyses by (**a**,**b**) changing the concentration of TMB (0.15 mM, 0.2 mM, 0.25 mM, 0.3 mM, 0.35 mM, 0.4 mM) while keeping H_2_O_2_ concentration (20 mM) constant and (**c**,**d**) changing the concentration of H_2_O_2_ (10 mM, 20 mM, 40 mM, 60 mM, 80 mM, 100 mM, 120 mM) while keeping TMB concentration (200 µM) constant. The concentration of Cu^2+^ was 50 µM.

In order to further study the peroxidase activity of Cu(II) ions, the steady-state kinetic parameters of the reaction between H_2_O_2_ and TMB were investigated. The kinetic data were calculated on the basis of Lineweaver–Burk plots: $\frac{1}{V}=\frac{K_{m}}{V_{max}\left[S\right]}+\frac{1}{V_{max}}$, where *V* is the initial velocity, *S* is the substrate concentration, *V_max_* is the maximum velocity, and *K_m_* is the Michaelis constant [28]. As shown in Figure 2a,c, the initial reaction rate accelerated with the increasing concentration of TMB or H_2_O_2_. The Michaelis–Menten constant (*K_m_*) and the maximum initial reaction rate (*V_max_*) are calculated from Lineweaver–Burk plots (Figure 2b,d). The *V_max_* values (1.69 and 1.46 × 10^−7^) of Cu(II) ions toward TMB and H_2_O_2_ are higher than that of horseradish peroxidase (HRP), indicating the high-speed catalytic efficiency of Cu(II) ions toward substrates. The reason should be attributed to the hypothesis that free Cu(II) ions can greatly promote the entry of substrate into the active site, thus promoting the catalytic rate. In comparison with HRP, the *K_m_* value (0.36 and 1.89 mM) toward substrates TMB and H_2_O_2_ [28] demonstrated the stronger binding affinity of Cu(II) ions, thus exhibiting superior catalytic performance. In virtue of low cost and comparable peroxidase catalytic activity, Cu(II) ions can be adopted as a potential substitute for nature peroxidase for appealing applications.

**Figure 3:**
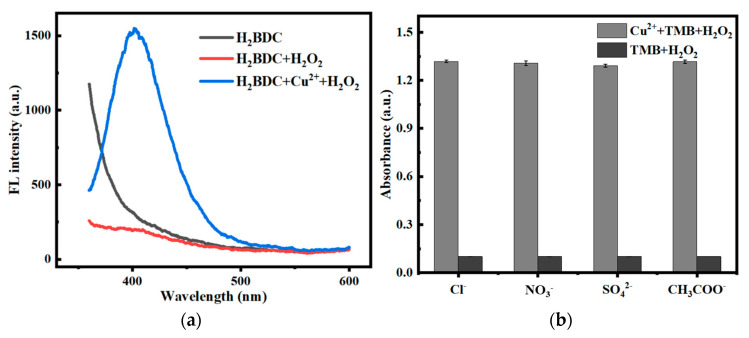

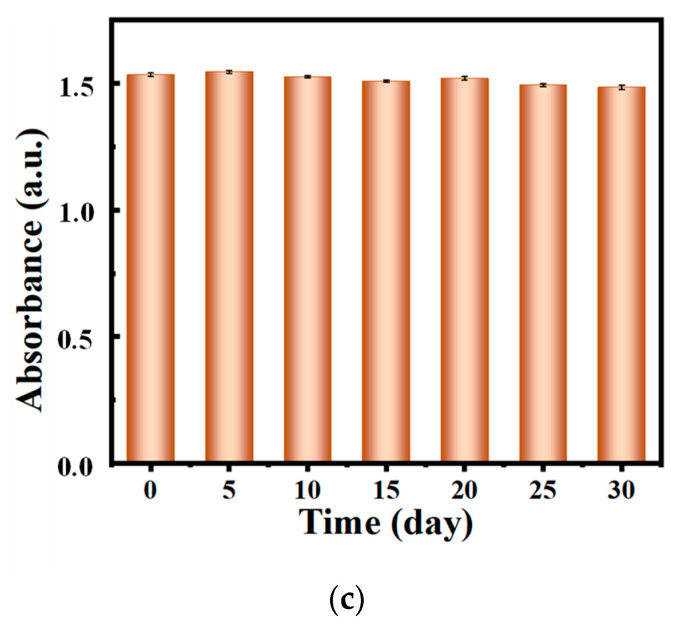
(**a**) Fluorescence spectra of H_2_BDC, H_2_BDC + H_2_O_2_, and H_2_BDC + H_2_O_2_ + Cu^2+^. (**b**) The effect of Cu(II) in copper salts with different anions (such as Cl^−^, NO_3_^−^, SO_4_^2−^, CH_3_COO^−^) on peroxidase-like activity. (**c**) The effect of storage time on the peroxidase-like activity of Cu(II) ions.

To uncover the catalytic mechanism, we investigated the active intermediates to confirm the peroxidase-mimicking activity of Cu(II) ions. Terephthalic acid (H_2_BDC) was used as a fluorescent agent for tracking hydroxyl radicals (•OH) [29]. Because of the peroxidase-mimicking activity of Cu(II) ions, H_2_O_2_ was catalytically oxidized to generate •OH. The •OH was trapped by H_2_BDC to form 2-hydroxy terephthalic acid (oxH_2_BDC), and oxH_2_BDC can exhibit a strong fluorescence with an excitation wavelength at 315 nm and emission peak at around 410 nm. As shown in Figure 3a, compared with H_2_BDC or the mixture of H_2_BDC and H_2_O_2_, only in Cu(II) ions system can exhibit fluorescent signals, which indicates the generation of •OH and oxH_2_BDC. It can be inferred that •OH is generated from the department of H_2_O_2_ by the peroxidase-mimicking activity of Cu(II) ions. Therefore, the possible catalytic mechanism was speculated through a Fenton-like reaction of Cu(II) ions.

We also studied the effect of Cu^2+^ in copper salts with different anions (such as Cl^−^, NO_3_^−^, SO_4_^2−^, and CH_3_COO^−^) on peroxidase activity, as shown in Figure 3b. The basically consistent absorbance implied the equivalent catalytic capacity of different copper salts toward substrates TMB and H_2_O_2_. Figure 3c shows the effect of storage time on the peroxidase activity of Cu(II) ions. Clearly, almost no changes in the catalytic capacity of Cu(II) ions were observed after storage for one month. From these results, we can conclude that Cu(II) ions possess an effective and stable intrinsic peroxidase activity.

### 2.2. Glyp Can Suppress Peroxidase-Mimicking Activity of Cu(II) Ions

**Figure 4:**
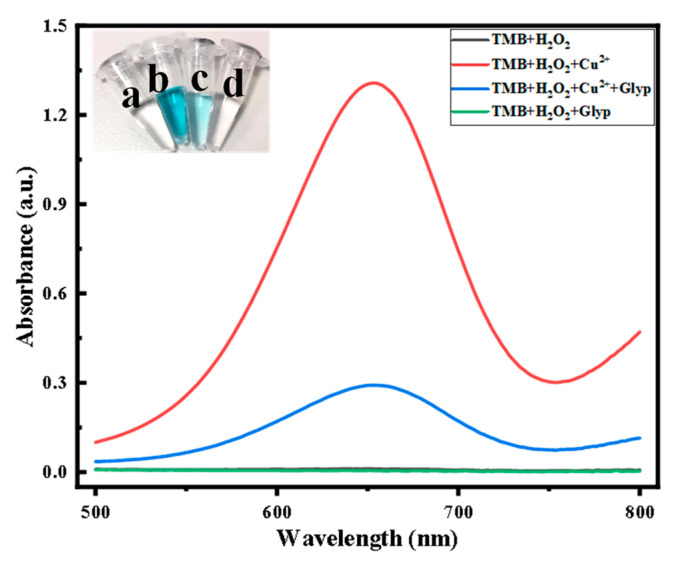
UV-vis spectra of TMB + H_2_O_2_ (a, black line), Cu^2+^ + TMB + H_2_O_2_ (b, red line), Cu^2+^ + TMB + H_2_O_2_ + Glyp (c, blue line), and TMB + H_2_O_2_ + Glyp (d, green line). The inset presents the corresponding photograph.

Cu^2+^ and Glyp can generate N-(phosphonomethyl) glycine copper (II) chelate, in which phosphate and carboxyl groups have very strong affinities toward Cu^2+^. With the generation of the Glyp–Cu^2+^ complex, the peroxidase activity of Cu^2+^ can be quenched. UV-vis absorption spectra were studied to demonstrate the feasibility of the developed strategy, as revealed in Figure 4. It can be seen that the appearance of Glyp itself has no effect on the TMB–H_2_O_2_ color reaction. When Cu^2+^ is pre-incubated with Glyp and added to the TMB–H_2_O_2_ system, the absorbance decreases, and the blue color of the solution fades. That is, the Glyp–Cu^2+^ complex has much lower catalytic power. This phenomenon indicated that Glyp did inhibit the peroxidase activity of Cu^2+^ by forming the Glyp–Cu^2+^ complex. These results verified the feasibility of using our designed suppression peroxidase activity of Cu(II) ions for the determination of Glyp.

### 2.3. Optimization of Experimental Conditions

**Figure 5:**
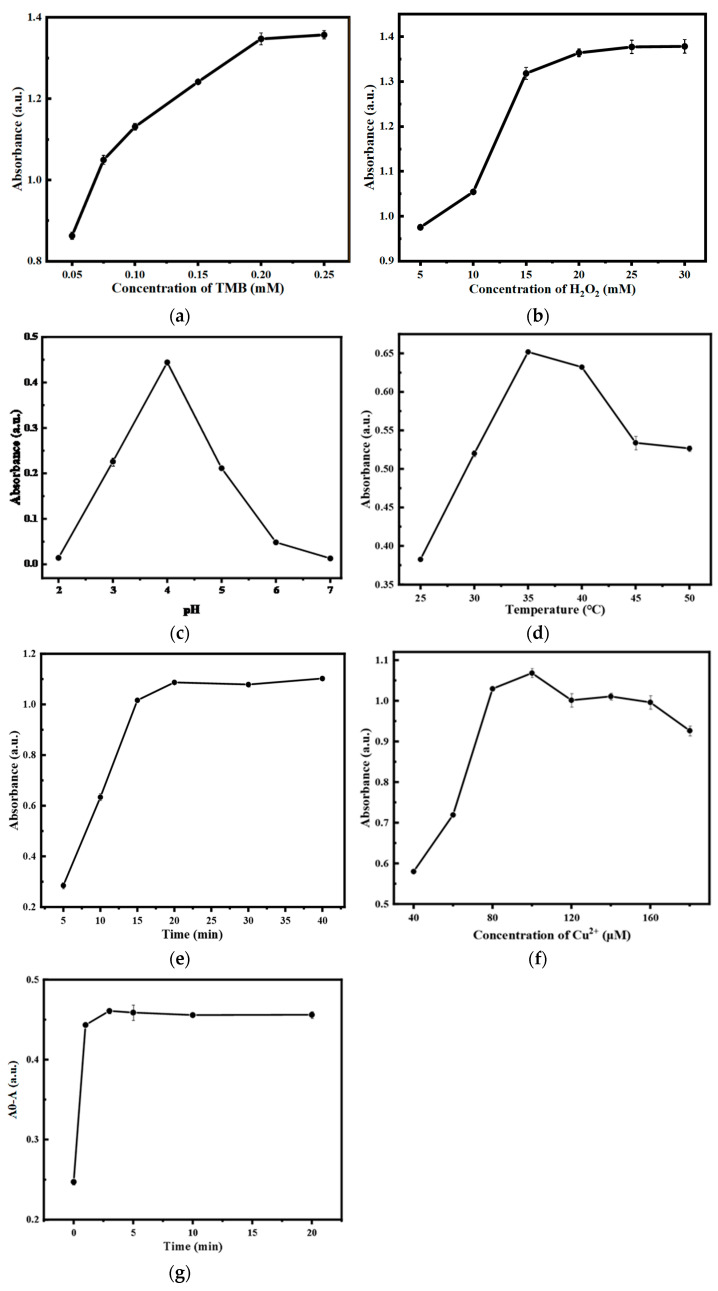
Optimization of concentration of TMB (**a**) and H_2_O_2_ (**b**), pH (**c**), temperature (**d**), and reaction time (**e**) for the catalytic activity of Cu^2+^. Optimization of concentration of Cu^2+^ (**f**) and the incubation time of Cu^2+^ and Glyp (**g**).

In order to obtain the best response performance of our proposed sensor, the influence of reaction conditions, including the concentration of TMB and H_2_O_2_, pH, reaction temperature, Cu^2+^ concentration, catalytic reaction time, as well as incubation time of Cu^2+^ and Glyp, were optimized. The catalytic activity of Cu^2+^ depends on the concentration of TMB and H_2_O_2_, as shown in Figure 5a,b. The absorbance value reached the plateau at 200 μM of TMB and 20 mM of H_2_O_2_ concentration, respectively, so the concentrations of TMB and H_2_O_2_ were fixed at 200 μM and 20 mM. Like the natural enzyme, the peroxidase-mimicking activity of Cu^2+^ is sensitive to pH value. Therefore, the pH was surveyed ranging from 2.0 to 7.0, as shown in Figure 5c. It can be obviously observed that the absorbance reached its maximum when pH was 4.0. Hence, we fixed the pH value at 4.0 for the following experiment. Temperature is another key factor in most enzymatic reactions. Figure 5d displays the effect on the catalytic activity of Cu^2+^ retained the optimal performance at 35 °C. To ensure a complete reaction, the effect of reaction time on the chromogenic reaction was investigated. Figure 5e presents the time dependence of absorbance, along with the extension of the catalytic reaction time, the absorbance increased and reached a constant level until 20 min, and therefore 20 min of catalytic reaction time was used. In light of the dependence of the concentration of the catalyzer, we then investigated the effect of Cu^2+^ concentration on the catalytic capacity of TMB + H_2_O_2_. As shown in Figure 5f, the absorbance value increased with the Cu^2+^ concentration initially, then remained constant while the concentration of Cu^2+^ was 100 μM. Therefore, 100 μM Cu^2+^ is selected for the catalytic reaction.

Finally, we also investigated the incubation time of Cu^2+^ and Glyp, as shown in Figure 5g. The absorbance decreased value Δ*A* = *A*_0_ − *A* (*A*_0_ and *A* are the absorption value of the system at 653 nm without and with Glyp, respectively) could reach reaction plateau within 3 min; therefore, 3 min was selected as the preincubation time of Cu^2+^ and Glyp.

### 2.4. Analytical Performance of Developed Strategy

**Figure 6:**
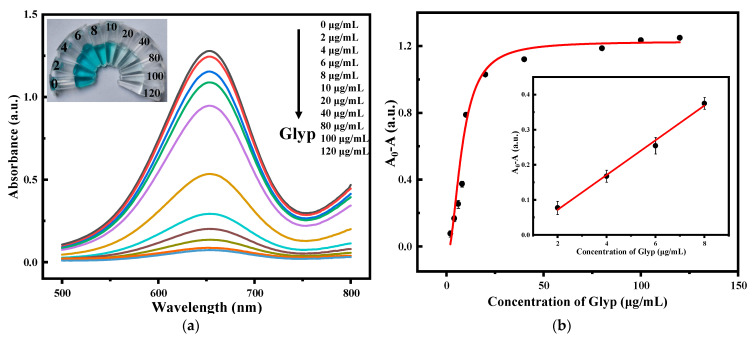
The colorimetric assay for Glyp. (**a**) UV-vis spectrum of the analytical platform response to different concentrations of Glyp. (**b**) Relationship between absorbance decreased value at 653 nm ΔA and Glyp concentration.

The catalytic capacity of Cu^2+^ toward TMB–H_2_O_2_ for the quantitative analysis performance was investigated after preincubation with various amounts of Glyp. As displayed in Figure 6a, the absorbance peak at 653 nm gradually decreased with the increment of Glyp concentration in the range of 2–120 μg/mL. As shown in Figure 6b, a suitable linear relationship was obtained between the absorbance decreased value Δ*A* and the Glyp concentration in the range of 2 to 20 µg/mL, and the fitted linear equation is expressed as Δ*A* = 0.02164*C_Glyp_* − 0.0058 with a coefficient of 0.99322, where *C_Glyp_* is the concentration of Glyp. The limit of detection (LOD) was determined to be 95.6 ng/mL using the principle of 3S/N (S: standard deviation of the blank groups, N: the slope of the standard curve). Such a low detection limit was comparable to and even better than most of the previous methods (Table 1) [30,31,32,33,34]. Therefore, the response performance of the developed strategy can fully satisfy the demand for Glyp detection in practice applications.

**Figure 7:**
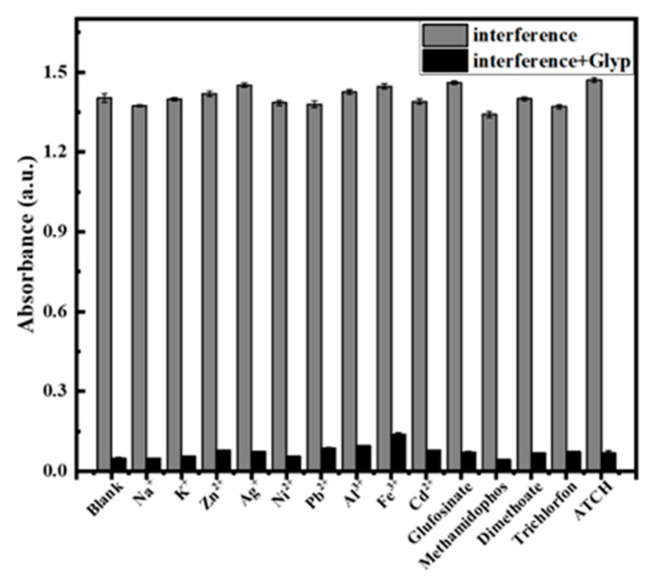
Selectivity evaluation of this colorimetric sensing system. The concentration of TMB, H_2_O_2_, Cu^2+^, Glyp, and interferences was 200 μM, 20 mΜ, 100 μM, 100 μg/mL, and 100 μg/mL, respectively.

The selectivity of the developed method was demonstrated by the determination of common metal ions such as Na^+^, K^+^, Zn^2+^, Ag^+^, Ni^2+^, Pb^2+^, Al^3+^, Fe^3+^, and Cd^2+^ in water. In addition, compounds, including organophosphorus pesticides, including glufosinate, methamidophos, dimethoate, trichlorion, and acetylthiocholine (ATCH), which are Glyp analogs, were also tested. The selectivity experiment was performed by comparing the absorbance value of interfering substances before and after adding Glyp. As shown in Figure 7, only Glyp coexistence with interfering substances can induce a significant absorbance peak decrease at 653 nm, while the presence of only interfering substances has negligible effects on the peroxidase-mimicking activity of Cu^2+^. This result proved that the colorimetric method based on Cu^2+^ has excellent selectivity for Glyp. This suitable selectivity can be ascribed to the fact that only Glyp can chelate with Cu^2+^ and suppress the peroxidase activity of Cu^2+^.

### 2.5. Glyp Detection in Real Samples

Having verified the response performance of this colorimetric assay, the practical utility of the developed method in real samples was assessed. Three varied contents of Glyp were spiked into the pretreated Xiangjiang River samples, and investigate the reliability of our developed method. As shown in Table 2, the recoveries were calculated to be in the range of 96.17–105.12%, and the relative standard deviations ranged from 2.36% to 5.15%, indicating that the colorimetric method provides acceptable accuracy and can be applied for rapid detection of Glyp in actual samples. We also explore HPLC as a reference method to determine the recoveries of real samples. Apparently, the results obtained from our method are in accordance with the HPLC analysis, which indicated that the proposed colorimetric assay provides high accuracy and reliability and holds a promising application for the estimation of Glyp in actual samples.

## 3. Materials and Methods

### 3.1. Chemicals and Instrument

Glyp, 3,3,5,5-tetramethylbenzidine (TMB, 99%), tri-(hydroxymethyl)-aminomethane, copper chloride dihydrate (CuCl_2_·2H_2_O), hydrogen peroxide (H_2_O_2_, 30wt%), anhydrous sodium acetate (CH_3_COONa), acetic acid (CH_3_COOH), and sodium hydroxide (NaOH) were ordered by Aladdin Reagent Co., Ltd. (Shanghai, China). Glufosinate, methamidophos (99%), trichlorfon, dimethoate, and acetylthiocholine (ATCH) were ordered from Tanmo Quality Inspection Technology Co., Ltd. (Changzhou, China).

Fluorescence analysis was carried out on an F7100 fluorescence spectrophotometer (Hitachi, Japan) with excited at 315 nm excitation, and the fluorescence emission range was recorded between 300 and 600 nm. UV-vis absorption spectrum was recorded on a Perkin-Elmer Lambda X50 UV-vis spectrometer.

### 3.2. Cu(II) Ions as a Peroxidase

Cu^2+^ in copper salts with different anions, including CuCl_2_·2H_2_O, Cu(NO_3_)_2_, CuSO_4,_ and Cu(CH_3_COO)_2,_ was used to investigate the peroxidase activity of Cu^2+^. A total of 10 μL 1 mM Cu^2+^, 10 μL HAc-NaAc buffer (0.1 mM, pH 4.0), 60 μL ultrapure water, 10 μL 2 mM TMB, and 10 μL 200 mM H_2_O_2_ were mixed evenly. Then, the catalytic oxidation reaction was carried out at 35 °C for 20 min, and the absorption spectrum was measured using Lambda 750s UV-vis Spectrometer (PerkinElmer, Waltham, MA, USA).

### 3.3. Optimization of the Detection Conditions

To investigate the optimal concentration of TMB, the reaction system contained 20 mΜ H_2_O_2_, 50 μM Cu^2+,^ and different concentrations of TMB. While fixed at the amount of 200 μM TMB, 50 μM Cu^2+^ was incubated with different concentrations of H_2_O_2_. To investigate the optimal concentration of Cu^2+^, different concentrations of Cu^2+^ were incubated. The reaction system contained 200 μM TMB and 20 mΜ H_2_O_2_. To investigate the optimal temperature for the catalytic activity of Cu^2+^, the reaction system contained 200 μM TMB, 20 mΜ H_2_O_2,_ and 50 μM Cu^2+^ was incubated in water bath pots at different temperatures.

### 3.4. Enzyme Kinetics Testing of Cu(II) Ions

The steady-state kinetic parameters of Cu(II) ions were measured under optimized experimental conditions using H_2_O_2_ and TMB as substrates. Under water bath conditions at 35 °C, in the presence of 10 μL H_2_O_2_ and 10 μL TMB, 10 μL 1 mM Cu^2+^ was mixed in 10 μL HAc-NaAc buffer (0.1 mM, pH 4.0) and 60 μL ultrapure water. During testing, change the TMB concentration (0.1 mM, 0.15 mM, 0.2 mM, 0.25 mM, 0.3 mM, 0.35 mM, 0.4 mM) while maintaining 200 mM H_2_O_2_, or change the H_2_O_2_ concentration (10 mM, 20 mM, 40 mM, 60 mM, 80 mM, 100 mM, and 120 mM) while maintaining 2 mM TMB. In the kinetic testing mode of the UV visible spectrophotometer, the absorbance values were tested every 1 min at a wavelength of 653 nm, and the corresponding kinetic parameters were obtained by continuously testing for 5 min.

### 3.5. Mechanism of Cu(II) Catalyzed Reaction

In order to study the catalytic reaction mechanism of Cu(II) ions, terephthalic acid (H_2_BDC) was used as a fluorescent agent for tracking hydroxyl radicals (•OH) in the Cu^2+^–H_2_O_2_ system, producing 2-hydroxy terephthalic acid with an excitation wavelength at 315 nm and emission peak at around 410 nm. A total of 10 μL 1 mM Cu(II) ions, 40 μL 200 mM H_2_O_2,_ and 10 μL PTA (4 mmol/L) were added at room temperature and maintained a total volume of 100 μL. The final mixture was monitored by the fluorescence intensity at different reaction systems on a fluorescence spectrophotometer.

### 3.6. Detection of Glyp

A total of 10 μL HAc-NaAc buffer (0.1 mM, pH 4.0), 50 μL ultrapure water, 10 μL 1 mM Cu^2+^, and 10 μL different concentrations of Glyp were pre-mixed together and incubated for 3 min. Then, 10 μL 2 mM TMB and 10 μL 200 mM H_2_O_2_ were added to the above mixture and reacted at 35 °C for 20 min. Finally, UV-vis spectra were recorded.

### 3.7. Selectivity Study for Glyp Detection

The selectivity of the developed method was demonstrated by the determination of common metal ions such as Na^+^, K^+^, Zn^2+^, Ag^+^, Ni^2+^, Pb^2+^, Al^3+^, Fe^3+^, and Cd^2+^ in water. In addition, compounds, including organophosphorus pesticides, including glufosinate, methamidophos, dimethoate, trichlorion, and acetylthiocholine (ATCH), which are Glyp analogs, were also tested. The final concentration of TMB, H_2_O_2_, Cu^2+^, Glyp, and interferences was 200 μM, 20 mΜ, 100 μM, 100 μg/mL, and 100 μg/mL. The reaction system is the same as above.

### 3.8. Determination of Glyp in Real Samples

The application of this method in the actual environment was further studied by taking the Xiangjiang River water. The water sample was filtered using a microporous cellulose membrane (0.22 μm) to remove insoluble impurities. Various contents of Glyp standard solution were added into the pretreated sample solution for determination. Then, Cu^2+^, TMB, and H_2_O_2_ were added in turn. Signal acquisition was the same as the steps described above for detecting Glyp.

For HPLC analysis, the sample was filtered and purified by adding trisodium citrate under acidic conditions. The Glyp in the sample reacted with a 9-fluoromethyl chloroform ester to generate fluorescent products. The derivative by-products can be removed by liquid–liquid extraction with dichloromethane. The sample to be tested was detected by liquid chromatography with a fluorescence detector, and the concentration level of Glyp in the sample was determined by retention time qualitative analysis and external standard method quantitative analysis. The C18 column (Agilent TC-C18, 250 mm × 4.6 mm, 5.0 μm) was used as a stationary phase. The excitation wavelength was 254 nm, and the detection wave was 302 nm in length. The phosphoric acid solution was used as a mobile phase with a 1.000 mL/min flow rate, and acetonitrile elution was applied at room temperature. The sample injection volume was 10 µL, and the retention time for Glyp was 8.348 min. for HPLC analysis. The stock solution (1000 mg/L) of Glyp was prepared in the mobile phase, and different Glyp concentrations between 10.00 mg/L and 1.00 mg/L were applied to obtain the calibration curve of HPLC analysis. To perform HPLC analysis, samples were filtered with a syringe filter (0.22 μm), which was stored in a refrigerator at −20 °C in the dark before use.

## 4. Conclusions

In summary, we successfully established a facile and efficient colorimetric method for the determination of glyphosate via suppression of peroxidase activity by Cu^2+^. The formation of the glyphosate–Cu^2+^ complex consequently hindered the catalytic capacity of Cu^2+^. The proposed method reported a simple and quick quantitative assay of glyphosate concentration in the linear range of 2–20 μg/mL and LOD of 95.6 ng/mL, which exhibit a suitable analysis performance of the developed strategy for glyphosate. Suitable recoveries were obtained in the detection of glyphosate in the actual sample. Further developments in this Cu^2+^ ions peroxidase strategy can be designed to activate response mode to overcome the potential impact of the negative signal way on the sensitivity. Furthermore, when faced with the analysis of glyphosate in complex environments, pretreatment of the sample can be carried out in advance to remove the possible interference effects by contaminants, such as the natural presence of Cu^2+^ ions or chelating agents. In the future, we plan to integrate a portable strip based on Cu^2+^ ions peroxidase in order to build a smartphone-based point-of-care platform for glyphosate determination. In virtue of its sensitivity and simplicity, our strategy will serve as a potential approach in the field of environmental analysis.

## Figures and Tables

**Table 1:** Response performance comparison of the proposed strategy with other methods for glyphosate detection.

Material	Detection Mode	Detection Range (μg/mL)	LOD (ng/mL)	Reference
Gold electrode	Electrochemical	--	270.51	30
Copper (II)- pyrocatechol violet based GlyPKit	Colorimetry	1.69~10.13	422	31
Silver Nanoparticles	UV-Vis	--	1000	32
	Raman transduction		1300	
Red-Emitting Polymerizable Guanidinium Dyes	Fluorometry	--	101.44	33
Molecularly imprinted polypyrrole	Voltametric	0.4~1.2	92	34
The suppression peroxidase-mimicking activity of Cu^2+^	Colorimetry	2~20	95.6	This work

**Table 2:** Determination of the recovery results for Glyp in water sample (n = 3).

Method	Added (μM)	Found (μM)	Recovery (%)	RSD (n = 3)
This work	2.5	2.615	104.60	5.13%
	3.0	2.885	96.17	2.36%
	6.0	6.307	105.12	3.58%
HPLC	2.5	2.530	101.20	0.86%
	3.0	2.986	99.53	0.79%
	6.0	6.013	100.22	0.82%

## Data Availability

Not applicable.
